# Wnt/β-catenin signaling integrates patterning and metabolism of the insect growth zone

**DOI:** 10.1242/dev.112797

**Published:** 2014-12-15

**Authors:** Georg Oberhofer, Daniela Grossmann, Janna L. Siemanowski, Tim Beissbarth, Gregor Bucher

**Affiliations:** 1 Department of Evolutionary Developmental Biology, Johann Friedrich Blumenbach Institute of Zoology and Anthropology, Georg-August-University, Justus von Liebig Weg 11, Göttingen D-37077, Germany; 2 Department of Medical Statistics, University Medical Center Göttingen, Humboldtallee 32, Göttingen D-37073, Germany

**Keywords:** Head, Growth zone, Segment addition zone, Wnt, Hedgehog, Gut, Senseless

## Abstract

Wnt/β-catenin and hedgehog (Hh) signaling are essential for transmitting signals across cell membranes in animal embryos. Early patterning of the principal insect model, *Drosophila melanogaster*, occurs in the syncytial blastoderm, where diffusion of transcription factors obviates the need for signaling pathways. However, in the cellularized growth zone of typical short germ insect embryos, signaling pathways are predicted to play a more fundamental role. Indeed, the Wnt/β-catenin pathway is required for posterior elongation in most arthropods, although which target genes are activated in this context remains elusive. Here, we use the short germ beetle *Tribolium castaneum* to investigate two Wnt and Hh signaling centers located in the head anlagen and in the growth zone of early embryos. We find that Wnt/β-catenin signaling acts upstream of Hh in the growth zone, whereas the opposite interaction occurs in the head. We determine the target gene sets of the Wnt/β-catenin and Hh pathways and find that the growth zone signaling center activates a much greater number of genes and that the Wnt and Hh target gene sets are essentially non-overlapping. The Wnt pathway activates key genes of all three germ layers, including pair-rule genes, and *Tc-caudal* and *Tc-twist*. Furthermore, the Wnt pathway is required for hindgut development and we identify *Tc-senseless* as a novel hindgut patterning gene required in the early growth zone. At the same time, Wnt acts on growth zone metabolism and cell division, thereby integrating growth with patterning. Posterior Hh signaling activates several genes potentially involved in a proteinase cascade of unknown function.

## INTRODUCTION

The Wnt/β-catenin (or canonical Wnt) and hedgehog (Hh) signaling pathways play important roles in animal pattern formation (Martin and Kimelman, 2009; Pires-daSilva and Sommer, 2003). Their involvement in posterior patterning appears to be an ancestral feature in animals. The Hh ligand is expressed in posterior tissues in short germ insects and in several annelids and functional work has revealed that in both clades it is required for segment maintenance but not segment establishment (Dray et al., 2010; Farzana and Brown, 2008; Seaver and Kaneshige, 2006). The requirement of the Wnt/β-catenin pathway for posterior growth and elongation has been identified in vertebrates (Martin and Kimelman, 2008; Shimizu et al., 2005) and protostomes. For instance, it is essential for posterior development in arthropods (Bolognesi et al., 2008; McGregor et al., 2008; Miyawaki et al., 2004) and posterior expression of Wnt ligands is found in onychophorans and annelids (Hogvall et al., 2014; Janssen et al., 2010). Although the conserved involvement of these pathways in posterior patterning is now well established, it has been proposed that a comprehensive identification of their respective target gene sets is required in order to assess the degree of similarity of the gene regulatory networks downstream of the Hh and Wnt/β-catenin pathways (Martin and Kimelman, 2009). Such a comprehensive identification of Wnt and Hh target gene sets of any growth zone is currently lacking for any protostome.

Within arthropods, embryonic patterning has been most thoroughly studied in the fruit fly *Drosophila melanogaster*, in which all segments form almost simultaneously at the syncytial blastoderm stage. In this long germ mode of embryogenesis, the nuclei are not yet separated by cell membranes, which allows for diffusion of transcription factors between nuclei, thereby obviating the need for signaling pathways (St Johnston and Nüsslein-Volhard, 1992). By contrast, most insects show the short germ mode of embryogenesis in which segmentation occurs from a posterior elongation and differentiation zone in a fully cellularized environment. [We use the traditional term ‘growth zone’ (GZ), although most of the early elongation in *Tribolium* is actually attributed to convergent extension (Sarrazin et al., 2012); note that in other protostomes, the term ‘segment addition zone’ is used instead.] Likewise, head patterning in short germ embryos occurs after cellularization (Benton et al., 2013; Handel et al., 2000; Posnien et al., 2010; Tautz et al., 1994). Therefore, signaling pathways are expected to play a more fundamental role in ancestral insect patterning compared with *Drosophila*.

The red flour beetle *Tribolium castaneum* has become a major model for short germ embryogenesis. Several signaling pathways contribute to *Tribolium* patterning: FGF signaling is required for aspects of extraembryonic and embryonic development (Sharma et al., 2013), while torso signaling is required for the establishment of the GZ (Schoppmeier and Schröder, 2005). Wnt and Hh signaling play several subsequent roles in early embryogenesis in *Tribolium* and show corresponding dynamic expression. First, during axis formation, Wnt/β-catenin signaling is required for posterior development and needs to be repressed to allow anterior development, as in vertebrates (Bolognesi et al., 2008; Fu et al., 2012). In a second phase, *Tc-hh* and *Tc-wg* expression arise *de novo* in the head anlagen, while Wnt and Hh signaling remain active in the posterior, where Wnt signaling is needed for elongation of the GZ in *Tribolium* and other arthropods and for the expression of some pair-rule genes in *Tribolium* (Beermann et al., 2011; Bolognesi et al., 2008) (see supplementary material Fig. S1 and Fig. 7). Subsequently, the interaction of adjacent *hh* and *wg* activity maintains parasegment boundaries in the trunk – a function conserved in protostomes (Damen, 2002; Dray et al., 2010; Farzana and Brown, 2008; Oppenheimer et al., 1999; Wohlfrom et al., 2006).

At early stages of embryogenesis, Wnt and Hh signaling appear to differ between *Tribolium* and *Drosophila*. In the early *Tribolium* head anlagen, *Tc-wg* and *Tc-hh* form adjacent stripes from early germ rudiment stages onwards, resembling a bona fide ocular parasegment boundary (Farzana and Brown, 2008; Nagy and Carroll, 1994; Posnien et al., 2011). In *Drosophila*, by contrast, the early ocular domain of *wg* is not a stripe but a more extensive ‘head blob’, and *hh* arises somewhat later. The latter initially forms a broad stripe, which overlaps with the head blob dorsally and extends to the ventral embryo without contacting *wg* expression there (Ingham and Hidalgo, 1993; Tabata et al., 1992). Parasegment boundary-like expression in ocular and antennal segments arises only later, at approximately stage 10, by splitting of the head blob and the *hh* domains (Gallitano-Mendel and Finkelstein, 1997; Tabata et al., 1992; Ntini and Wimmer 2011). In the *Tribolium* GZ, adjacent *Tc-wg* and *Tc-hh* expression domains are found before and throughout elongation. Likewise, *wg* is expressed in a posterior domain from early blastoderm stages onward in *Drosophila* but the corresponding *hh* domain arises later, concomitant with the trunk stripes.

Hence, there appears to be two early Wnt and Hh signaling centers in *Tribolium*, which have no direct correlate in *Drosophila* at corresponding early stages. Further, they are located in two parts of the embryo, which form differently in *Drosophila*, namely the anterior head and GZ (Posnien et al., 2010; Tautz et al., 1994). Interestingly, the position of the head signaling center corresponds to the vertebrate midbrain-hindbrain boundary and several head patterning genes are activated in stripes parallel to this boundary in *Tribolium* (Posnien et al., 2011; Urbach, 2007). The expression of *hh* and *wg* in the hemimetabolous insect *Gryllus bimaculatus* is similar to that in *Tribolium*, suggesting that this represents the ancestral situation (Miyawaki et al., 2004).

The interactions of the Wnt/β-catenin and Hh pathways at these putative signaling centers of short germ embryos have not been studied to date, nor have their target gene sets been determined. We set out to reveal their genetic interactions and to reveal the respective target gene sets by RNA-seq after RNAi at the germ rudiment stage in order to provide a comprehensive picture of Wnt/β-catenin and Hh signaling in the GZ and head. We found that, unlike in *Drosophila*, the Hh pathway acts upstream of the Wnt/β-catenin pathway in the head, whereas in the GZ Wnt/β-catenin activated Hh expression. Unexpectedly, we did not find many target genes for the head signaling center, whereas both pathways had significant sets of non-overlapping target genes in the GZ. Ribosomal proteins and posterior patterning genes, including *Tc-caudal*, *Tc-hairy*, *Tc-odd-skipped* (*odd*) and *Tc-even-skipped* (*eve*), were regulated by Wnt/β-catenin signaling. Further, the mesodermal regulator *Tc-twist* and several hindgut specification genes were Wnt dependent. Hence, Wnt/β-catenin signaling acts on all three germ layers in the GZ. Hh signaling activated genes that are potentially involved in a proteolytic cascade. Finally, we revealed an unexpected essential role of the Wnt target gene *Tc-senseless* (*Tc-sens*) in hindgut formation.

## RESULTS

### Complementary interactions of the Wnt/β-catenin and Hh pathways in the head and GZ

**Fig. 1.**
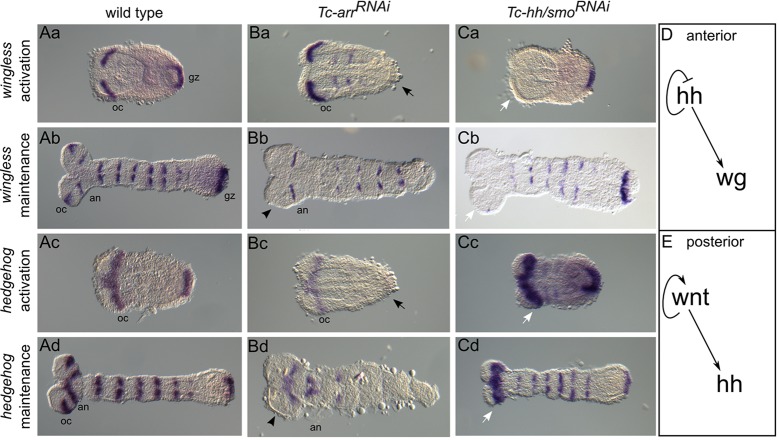
**Complementary interactions of Wnt and Hh signaling in the head and GZ.** (A-C) Expression of *Tc-wg* (rows 1 and 2) and *Tc-hh* (rows 3 and 4) in wild-type (Aa-d) and RNAi-treated embryos with interrupted Wnt (Ba-d) or Hh (Ca-d) signaling in germ rudiments (rows 1 and 3) and elongating germ bands (rows 2 and 4). Anterior is oriented to the left. (Ba-d) When the Wnt pathway was disrupted, both *Tc-wg* and *Tc-hh* expression was abolished in *Tc-arr^RNAi^* embryos in the GZ (black arrows) and at the ocular parasegment boundary (black arrowheads). (Ca-d) When the Hh pathway was disrupted, *Tc-wg* expression was missing in the anterior head of *Tc-hh^RNAi^* embryos (Ca,b, white arrows), and *Tc-hh* expression was largely present in *Tc-smo^RNAi^* but the anterior head domains appeared enlarged (Cc,d, white arrows). (D) Genetic interactions in the anterior head: Hh signaling acts upstream of *Tc-wg*. (E) Genetic interactions in the GZ: Wnt/β-catenin acts upstream of *Tc-hh* and shows autoregulation. Arrows indicate activation. an, antennal stripe; gz, growth zone; oc, ocular stripe.

We first analyzed the interaction of the pathways in germ rudiments [10-11 h after egg laying (AEL)] and elongating germ bands (12-15 h AEL). The expression of *Tc-wg* and *Tc-hh* was examined in embryos with disrupted Wnt/β-catenin signaling [*Tc-arrow* (*Tc-arr*)*^RNAi^*, Fig. 1Ba-d] or disrupted Hh signaling [*Tc-hh^RNAi^*, Fig. 1Ca,b; *Tc-smoothened* (*Tc-smo*)*^RNAi^*, Fig. 1Cc,d]. Strikingly, interactions were complementary: Hh signaling acted upstream of *Tc-wg* expression in the head (Fig. 1Ca,b,D), whereas Wnt/β-catenin signaling controlled *Tc-hh* expression in the GZ (Fig. 1Bc,d,E). Autoregulation was apparent only for the Wnt/β-catenin pathway in the GZ (Fig. 1Ba,b). Wnt/β-catenin signaling is also needed to maintain the ocular but not the antennal *Tc-hh* stripe (Fig. 1Bd, arrowhead). Autoinhibition of Hh signaling was found in the head (Fig. 1Cc,d, arrows). At the latest stages analyzed here, both *Tc-wg* and *Tc-hh* segmental trunk stripes start degenerating in the absence of Wnt signaling (Fig. 1Bb,d) but are not yet strongly affected in Hh knockdown (Fig. 1Cb,d). This is in line with previous findings that *Tc-engrailed* stripes appear to degenerate more quickly after knockdown of Wnt signaling as compared with Hh signaling (Bolognesi et al., 2009).

### RNA-seq is effective in revealing region-specific target gene sets

**Fig. 2.**
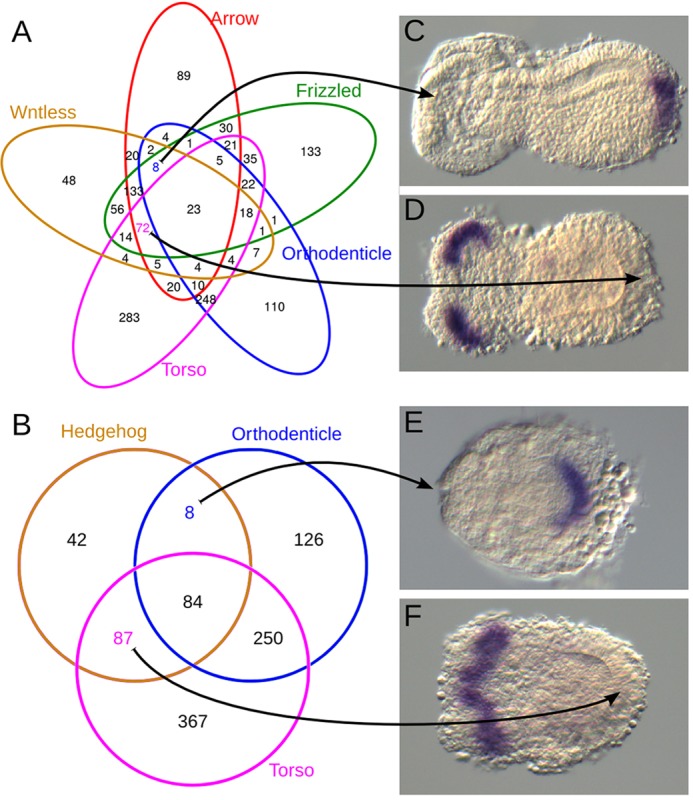
**Region-specific target gene sets identified by RNA-seq after RNAi.** (A,B) Venn diagrams showing the number of downregulated genes in the different RNAi treatments (colored circles) and their intersects. (C-F) In order to distinguish between anterior and posterior target gene sets, the ocular domains were deleted by *Tc-otd^RNAi^* (C,E) and the GZ expression domains were deleted by *Tc-tor^RNAi^* (D,F).

**Fig. 3.**
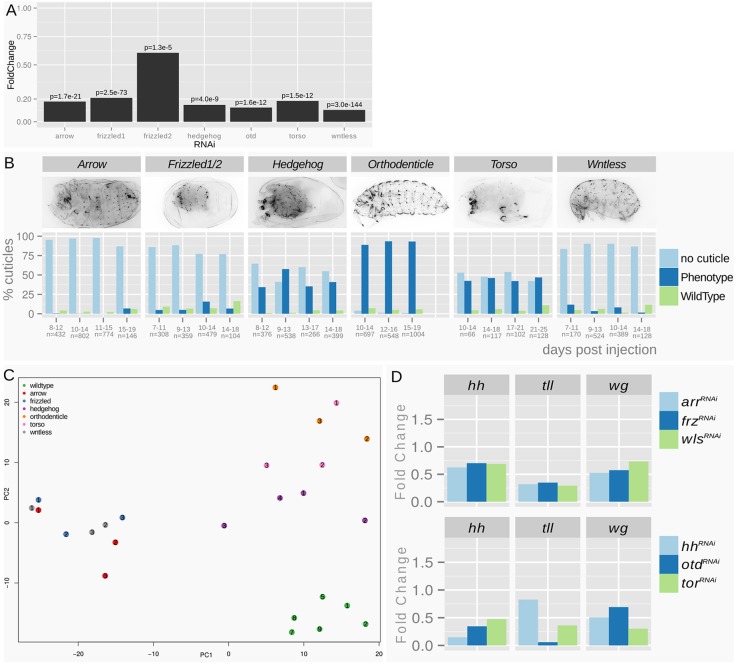
**Quality controls of the RNA-seq experiment.** (A) RNAi treatments resulted in a transcript reduction of 80-90% for the single knockdowns. In the double RNAi, *Tc-frizzled1* is reduced by 80% and *Tc-frizzled2* by 40%. Shown are fold changes of transcript levels compared with the wild type, with Benjamini Hochberg adjusted *P*-values (false discovery rate). (B) Quantitative cuticule phenotype analysis of siblings of the sequenced animals taken from the same batch. The penetrance of the treatment is shown by the high portion of phenotypic animals (‘no cuticle’ phenotypes derive from severely affected animals that stop embryogenesis prior to cuticle formation). Specificity of the treatment is shown by the cuticle analysis, where the expected morphological phenotype was observed. The analysis was repeated on subsequent days showing the persistence of the RNAi effect. (C) PCA of wild-type and knockdown samples confirms clustering of the treatments. (D) Fold change values of target genes with anterior and posterior expression domains. Note that *Tc-hh* is reduced to ∼10% in the Hh pathway knockdown but only to ∼70% in the Wnt pathway knockdowns. Assuming that *Tc-hh* expression is evenly distributed between head and GZ domains and that Wnt knockdown does not interfere with *Tc-hh* head expression, the *Tc-hh* knockdown of the GZ would be to ∼40% of the wild-type expression.

In order to reveal the respective target gene sets, we knocked down pathway components and identified the genes downregulated in germ rudiments (10-11 h AEL, Fig. 1Aa) by comparing their transcript levels with those in wild-type controls. The Hh pathway was disrupted by *Tc-hh^RNAi^* and Wnt/β-catenin signaling was suppressed by *Tc-arr^RNAi^*. In order to reduce the resulting large candidate gene set for the Wnt pathway, we added *Tc-frizzled1/2^RNAi^* and *Tc-wntless^RNAi^* treatments. *Tc-frizzled1/2^RNAi^* also targets the planar cell polarity (PCP) pathway and *Tc-wntless^RNAi^* affects all Wnt ligand signaling. The intersect of all three Wnt treatments was considered to contain targets of the Wnt/β-catenin pathway. These treatments cannot distinguish between anterior versus posterior target genes of a given pathway. Therefore, we included treatments in which either the head [*Tc-orthodenticle* (*Tc-otd*)*^RNAi^*] or the GZ [*Tc-torso* (*Tc-tor*)*^RNAi^*] *Tc-wg* and *Tc-hh* domains were depleted (Fig. 2C-F) (Schinko et al., 2008; Schoppmeier and Schröder, 2005). This allowed for the identification of head-specific versus GZ-specific target genes. For example, those *Tc-hh* targets that were additionally downregulated in *Tc-tor^RNAi^* but not in *Tc-otd^RNAi^* were considered exclusive posterior targets of Hh signaling (Fig. 2B,F; see Materials and Methods and Fig. 3 for details and controls).

Both pathways are thought to predominantly activate gene expression. Therefore, we primarily expected downregulation of highly expressed target genes. Nevertheless, we also checked the upregulated gene sets (supplementary material Table S3) and found that a high portion was expressed at very low levels in the wild type (70/78% of the upregulated posterior Wnt/Hh targets were expressed at low level compared with 8/16% in the respective downregulated gene sets; cutoff: read count <200; supplementary material Fig. S2). Hence, despite significant upregulation, their expression was still low. As we did not expect to find significant biological function for such low-level expressed genes and because the pathways are known to predominantly activate gene expression, we focused on the downregulated gene sets in the subsequent analysis.

**Fig. 4.**
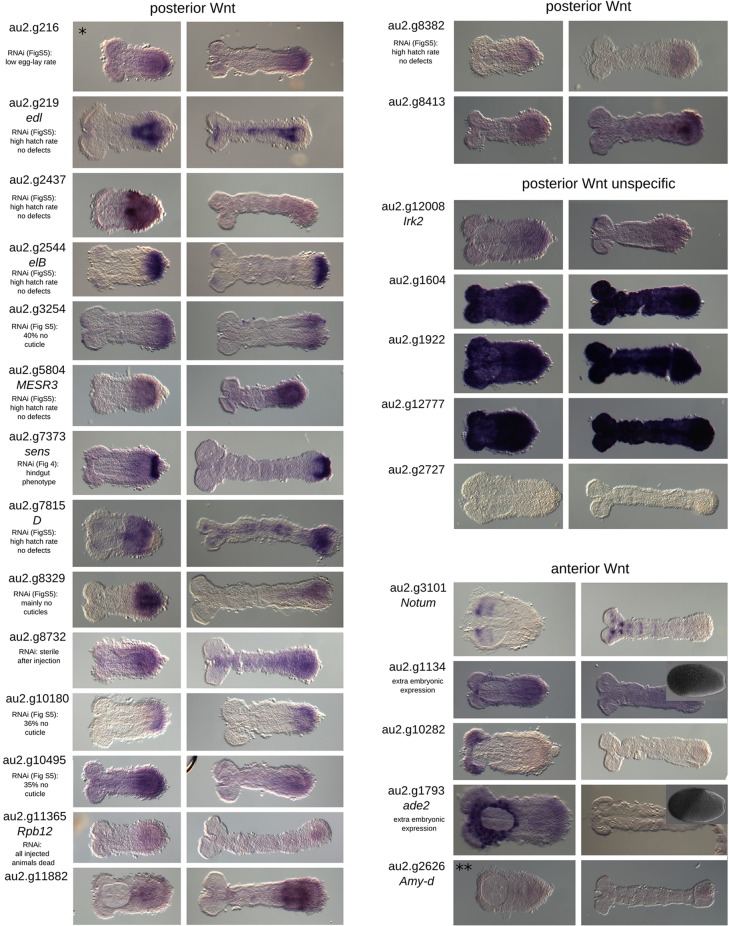
***In situ* hybridization of Wnt target candidates.** Posterior Wnt targets: 23 candidate genes were selected for evaluation in the *in situ* screen. We obtained clones for 21 of them. These included seven genes with a *Drosophila* ortholog or homolog known to be involved in signaling and that are transcription factors. Six of these candidates showed specific posterior expression in the early germ band rudiment. In addition, 14 genes with no *Drosophila* homolog were added to the candidates for the *in situ* screen. Ten showed posterior expression. Three genes with highly similar sequences related to a retrotransposon gave ubiquitous staining and one gene did not stain at all. In summary, of the 28 candidates (seven already published, seven with *Drosophila* homologs and 14 unknown) that we examined, 23 (82%) are expressed at the posterior in *Tribolium.* Gene au2.g216 (asterisk) is an overlapping candidate from both the Wnt and Hh gene sets. Anterior Wnt targets: this gene set contains eight genes. We selected six for the *in situ* screen and obtained clones for five. *Tc-notum* and au2.g10282 are expressed exclusively in the head at this early stage. *Tc-adenosine2* and au2.g1134 are expressed in the extraembryonic region at the anterior. *Tc-amylase distal* showed no expression. *Tc-ey* was known to be expressed in the head. Of the six genes (one published, three with *Drosophila* homologs and two unknown) that we examined, three are localized in the anterior germ band (50%) and two in the anterior extraembryonic region (33%). *Tc-amylase distal* (double asterisk) is an overlapping candidate from both the Wnt and Hh posterior gene sets. Insets show anterior expression at the blastoderm stage.

**Fig. 5.**
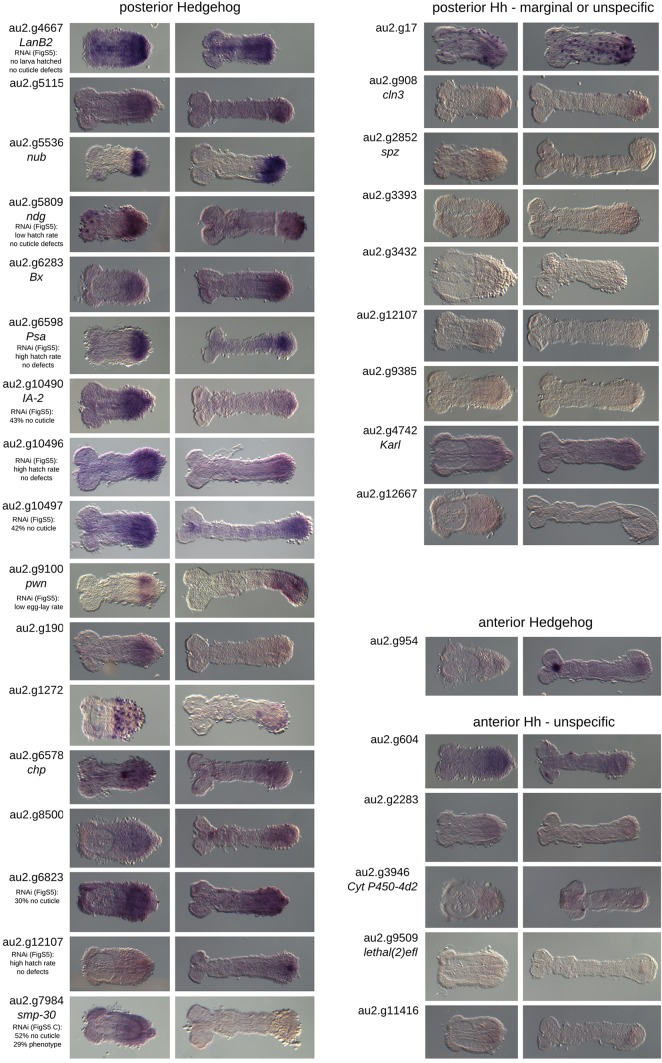
***In situ* hybridizations of candidate Hh targets.** Posterior Hh candidates: 27 genes were selected for the *in situ* screen, including 12 with a homologous gene in *Drosophila* and 15 without. Among the genes with a homolog, nine showed posterior expression. In the group of unknown genes, eight were expressed in posterior regions. In summary, we looked at 27 genes with 17 showing posterior expression (63%). Anterior Hh candidates: this set contains eight genes. Three had a homologous gene in *Drosophila.* We used seven in the *in situ* screen with only one showing anterior expression.

In order to validate our approach, we performed *in situ* hybridization with a subset of the identified target genes. Owing to our focus on pattern formation, we tested all transcription factors and signaling molecules. In order to potentially identify novel gene functions, we additionally tested all genes without an ortholog in *Drosophila*. However, we excluded genes with very low expression levels (below a normalized count of 200) because they were unlikely to produce a robust signal in an *in situ* hybridization. Twenty-eight of the 72 candidate targets of posterior Wnt signaling met our criteria. This set included seven genes that were already known to be expressed in the GZ in *Tribolium* (*Tc-brachyenteron*, *Tc-caudal*, *Tc-twist*, *Tc-eve*, *Tc-odd*, *Tc-hairy*, *Tc-ladybird*) (Berns et al., 2008; Brown et al., 1994; Cande et al., 2009; Choe et al., 2006; Schulz et al., 1998; Sommer and Tautz, 1993, 1994). Another 21 were examined by *in situ* hybridization and, indeed, 16 of these were specifically expressed in the GZ. The remaining five genes were either not expressed or were expressed ubiquitously (Fig. 4). In total, 23 of 28 genes showed posterior expression (82%). Five out of the eight genes in the anterior Wnt set were examined by *in situ* hybridization. Two were expressed in the head region, two in the anterior extraembryonic tissue, and one gave no signal. One additional gene [*Tc-eyeless* (*Tc-ey*)] was already known to be expressed in the head (Yang et al., 2009). Hence, 83% of the genes examined showed anterior expression. The posterior Hh set comprised 87 genes. We selected 27 candidates for *in situ* hybridization based on the same criteria and, of those, 17 showed posterior expression (63%). Seven out of eight anterior Hh targets were stained but only one showed anterior expression (14%) (Fig. 5). Overall, this demonstrated that our RNA-seq procedure reliably identified region-specific target genes, apart from the anterior Hh set.

### Distinct target gene sets of Hh and Wnt in the GZ

We found only a few target genes for the putative head signaling center. *Tc-notum* (a negative Wnt regulator) was found in the Wnt set as well as *Tc-ey*, in line with its known expression overlapping the *Tc-wg* domain (Giráldez et al., 2002; Yang et al., 2009). In the Hh set only au2.g954 showed specific anterior expression. As such a low number of targets was unexpected, we analyzed the individual read counts of candidate target genes in order to test whether these genes were missed in either treatment because they are synergistically regulated (supplementary material Fig. S3B). *Tc-lim1*, *Tc-otd1* and *Tc-ems* were not activated by either pathway. *Tc-sloppy paired 1* (*Tc-slp1*) and *Tc-ey* were regulated by both pathways indicating synergistic activation. However, two genes appeared to be regulated by Wnt but did not quite reach our conservative cutoff: *Tc-gsc* and *Tc-toy*. One further gene, *Tc-tll*, was sufficiently downregulated in the Wnt treatments to pass the cutoff. However, *Tc-tll* is expressed in both the head and GZ and, hence, is reduced in both *Tc-otd* and *Tc-tor* RNAi; therefore, it was neither part of the anterior-specific nor the posterior-specific gene sets. We confirmed regulation of *Tc-gsc* by Wnt and *Tc-ey* by both pathways (supplementary material Fig. S4) and regulation of *Tc-lim1*, *Tc-otd1* and *Tc-ems* by neither pathway by staining for their expression in knockdown embryos (supplementary material Fig. S5).

In the GZ, a similar number of targets was found for both pathways (Fig. 2). These gene sets were non-overlapping, apart from three genes (au2.g216, *Tc-SpdS*, au2.g2727; see gene IDs in supplementary material Tables S2 and S3)*.* Given that *Tc-hh* was activated by Wnt/β-catenin signaling in the GZ (see above), we had expected the Hh targets to be a subset of the Wnt target set. However, it is possible that the indirect knockdown of *Tc-hh* via *Tc-arr^RNAi^* was not efficient enough to knock down Hh target genes to the same degree as in the direct knockdown. Indeed, *Tc-hh* expression is knocked down to 10% in the *Tc-hh* RNAi treatment but to only 70% in the Wnt treatments (Fig. 3D). In order to further test this assumption, we released the cutoff for the fold change in the Wnt treatment from 0.5 to 0.7. This indeed led to 39 (44%) of the *Tc-hh* target genes appearing in the Wnt target gene set, supporting our hypothesis. In line with the overall correct segmentation of *Tc-hh^RNAi^* embryos (Farzana and Brown, 2008), only a single putative segmentation gene was found: *Tc-sloppy paired 2.* This gene has not been studied before and is a paralog of *Tc-slp1*, which is a secondary pair-rule gene (Choe and Brown, 2007; Maderspacher et al., 1998). Four genes were likely to be involved in signaling across cell membranes: *roadkill* (Hh), *cln3* (Notch, JNK), *CG10960* (Jak/Stat) and *Tc-spz1*, which is a member of the spätzle family (Toll) (Kent et al., 2006; Morisato and Anderson, 1994; Müller et al., 2005; Tuxworth et al., 2009). Interestingly, eight genes predicted to act as peptidases or peptidase inhibitors (*CG5618*, *CG5639*, *CG32473*, *Jonah65Aiii*, *Puromycin sensitive peptidase*, *calpainB*, *Serpin42Da*, *fat spondin*) were found (compared with none in the Wnt set) (Gelbart et al., 1997; Jékely and Friedrich, 1999). GO term analysis revealed no significantly enriched terms.

**Fig. 6.**
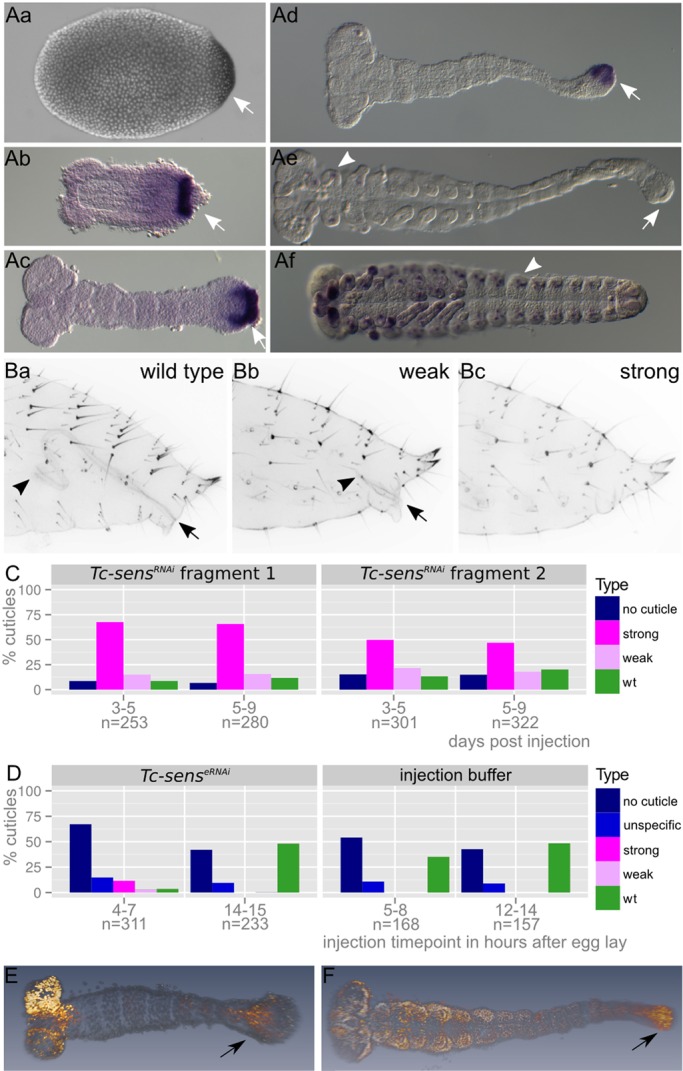
***Tc-sens* is required in germ rudiments for hindgut development.** (A) Expression of *Tc-sens* starts at the posterior pole in undifferentiated blastoderm embryos and is maintained in the GZ throughout elongation (arrows in Aa-d). During germ band retraction the posterior expression vanishes (arrow in Ae) and arises *de novo* in putative PNS precursors (arrowheads in Ae,f). (B) *Tc-sens^RNAi^* cuticle phenotype. Arrow and arrowhead mark extremes of hindgut. Weak phenotypes (Bb) show a shortened hindgut compared with wild type (Ba), while strong phenotypes lack the hindgut altogether (Bc). (C) Quantitation of phenotype classes in experiments with two non-overlapping dsRNA fragments. (D) Embryonic RNAi reproduced the parental RNAi phenotype when injected at 4-7 h AEL. Later injection (14-15 h AEL) did not elicit the hindgut phenotype, indicating an early essential function of *Tc-sens* in the GZ. The large proportion of ‘no cuticle’ and ‘unspecific’ phenotypes is due to artifacts of embryonic injection. (E,F) EdU cell proliferation assay in elongating (E) and elongated (F) germ bands. Proliferating cells are in orange (Alexa 488), nuclei are in gray (Hoechst 33342). The arrows indicate EdU marked cells in the GZ.

In the GZ gene set regulated by the Wnt pathway we found 21 ribosomal genes (whereas none was found in the Hh set). Several genes were involved in signaling pathways: *Tc-cAMP dependent PK1* (Hh), *elbowB* (Notch), *ETS-domain lacking* and *Misexpression suppressor of ras3* (Ras) (Baker et al., 2001; Huang and Rubin, 2000; Luque and Milán, 2007; Wang and Holmgren, 2000). Confirming previous results, we found *Tc-eve* (Beermann et al., 2011; Bolognesi et al., 2008; Bolognesi et al., 2009), but we also identified several additional posterior patterning genes to be Wnt/β-catenin targets: *Tc-caudal* and the pair-rule genes *Tc-odd* and *Tc-hairy* (Choe et al., 2006; Copf et al., 2004*;* Schulz et al., 1998; Sommer and Tautz, 1993). Further, we found *Tc-dichaete*, which in *Drosophila*, besides its role in neurogenesis and hindgut formation, is involved as an accessory factor in pair-rule gene regulation (Russell et al., 1996). The mesodermal genes *Tc-twist* (Sommer and Tautz, 1994) and *Tc-ladybird* (Cande et al., 2009; Jagla et al., 1997) were also found. Unexpectedly for this early embryonic stage, we found two genes with a known function in *Drosophila* hindgut formation: *Tc-brachyenteron* and *Tc-dichaete* (Berns et al., 2008; Sánchez-Soriano and Russell, 2000; Singer et al., 1996) and we identify *Tc-sens* as a novel player in gut development (see below). GO enrichment analysis using the *Drosophila* annotations included biological processes related to gastrulation, hindgut morphogenesis and transcription as well as biosynthesis and mitosis (supplementary material Table S1). EdU staining of elongating embryos confirmed enhanced cell division in the GZ at elongating stages in 15 out of 39 (38%) EdU-injected embryos (Fig. 6E,F). We do not detect much cell division at germ rudiment stages (not shown). This is in line with previous findings based on counting of cell divisions (Sarrazin et al., 2012).

### RNAi of selected candidate genes

The GZ gene sets included many posterior segmentation and hindgut formation genes. We searched for novel posterior patterning genes by knocking down selected genes specifically expressed in the GZ, including 13 from posterior Wnt targets, ten from posterior Hh targets and one gene found in both sets (supplementary material Fig. S6). Eight genes led to empty egg phenotypes, indicating strong early defects that lead to abortion of development prior to cuticle secretion (>30%, au2.g3254, au2.g6823, au2.g7815/*dichaete*, au2.g8329, au2.g10180, au2.g10490*/IA-2 ortholog*, au2.g10495, au2.g10497). Three genes led to low fertility or sterility (au2.g216, au2.g8732, au2.g9100/*pawn*). Two RNAis resulted in a very low hatch rate but without apparent cuticle defects (au2.g4667/*laminin B2*, au2.g5809/*nidogen*), in one treatment all injected animals died (au2.g11365/*Rpb12*) and one gene (au2.g7984/*senescence marker protein-30*) led to severe cuticle defects in which segments were malformed or missing and the bristle pattern was disturbed (supplementary material Fig. S6C).

### *Tc-sens* is required in early germ bands for hindgut formation

Unexpectedly, we found *Tc-sens* to be required for hindgut development (see supplementary material Fig. S7 for phylogenetic analysis). *Tc-sens* expression started at the posterior pole in blastoderm embryos. In early and elongating germ bands it was expressed in the GZ. Later, during germ band retraction, expression was lost in the GZ but started to arise in lateral spots, probably corresponding to the expression in the PNS observed in *Drosophila* (Nolo et al., 2000) (Fig. 6Aa-f). The first instar larval cuticles showed no defects after adult RNAi but the hindgut was missing or highly reduced (Fig. 6B,C). Morphologically, the hindgut becomes specified at a considerably later stage than that investigated here, raising the question of whether *Tc-sens* expression is required at the germ rudiment stage or later. For this reason we performed staged embryonic RNAi. Injections into early blastoderm stages (4-7 h AEL) interfered with gut formation, whereas injection into elongating embryos (14-15 h AEL, similar to the stage in Fig. 6Ac) did not elicit the gut phenotype (Fig. 6D). Apparently, *Tc-sens* is required early in the GZ for the specification of the hindgut anlage long before visible hindgut formation at the elongated germ band stage.

## DISCUSSION

### Complementary cross-regulation of Wnt and Hh pathways in head and trunk

The signaling centers that we investigated were predicted to play crucial roles in early patterning owing to their early onset of expression in a fully cellularized embryo. Importantly, the respective expression patterns differ from those in *Drosophila* but are similar to those in the hemimetabolous insect *G. bimaculatus* (Miyawaki et al., 2004), indicating that our results reflect the ancestral condition. We found a complementary cross-regulation of Wnt/β-catenin and Hh pathways in the anterior head and GZ: Hh signaling acted upstream of *Tc-wg* in the anterior head but had no influence on posterior GZ *Tc-wg* expression. In the GZ, by contrast, Wnt/β-catenin signaling acted upstream of *Tc-hh* and *Tc-wg* but Hh signaling had no influence on *Tc-wg* expression. Apparently, the interactions between these pathways differ in three embryonic regions, i.e. the anterior head, trunk and GZ. Different interactions have also been found in *Drosophila* head segments compared with the trunk but only the trunk interactions are conserved between these insects (Gallitano-Mendel and Finkelstein, 1997). The most surprising result was the upstream position of Hh signaling in the head because this differs from the *Drosophila* situation, in which ocular *wg* expression is unaffected in *hh* mutants, while the *hh* stripe is slightly altered in *wg* mutants (Gallitano-Mendel and Finkelstein, 1997). This corroborates previous evidence documenting the profound divergence of *Drosophila* head patterning (Kittelmann et al., 2013; Posnien et al., 2010).

### An unexpectedly low number of head target genes – synergism or delayed development?

**Fig. 7.**
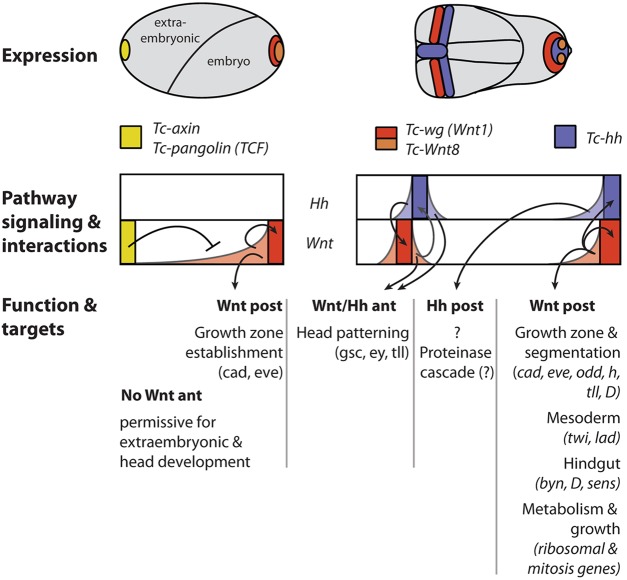
**Summary of early Wnt and Hh signaling functions in head and GZ.** The Wnt/β-catenin pathway has an early function in axis formation (left). During blastoderm stages, posterior Wnt/β-catenin signaling is required for GZ establishment and posterior patterning. Anterior repression of Wnt/β-catenin signaling is required for anterior development, with the extraembryonic serosa and the anterior embryo being most sensitive to elevated Wnt activity. Hh signaling does not appear to play a role at this stage. At the germ rudiment stage (right), posterior Wnt signaling maintains its own activity and initiates Hh signaling in the GZ. At the same time, adjacent stripes of *Tc-wg* and *Tc-hh* expression arise in the head anlagen, with Hh signaling being required for the initiation of *Tc-wg* expression. Later, mutual activation maintains this boundary. At this stage, we detected only a few target genes for both pathways in the head. The posterior Hh target gene set is largely non-overlapping with the Wnt set but its function remains elusive. Posterior Wnt/β-catenin signaling plays a central role in posterior development. First, it activates genes required for pattern formation in the GZ. This includes segmentation of the ectoderm, the formation of the mesoderm and the hindgut. Second, the expression of genes required for protein metabolism is enhanced by Wnt/β-catenin signaling. Finally, genes required for cell division are regulated by Wnt/β-catenin signaling. Hence, Wnt/β-catenin integrates patterning, metabolism and growth. Note that later functions of these pathways (such as parasegment boundary formation) are not depicted in this scheme and that the expression of further Wnt ligands has been omitted for simplicity. See text for further details. ant, anterior; post, posterior.

Our work identifies the first comprehensive target gene sets of the Hh and Wnt/β-catenin pathways in a short germ embryo. Our controls show that we faithfully identified region-specific genes regulated by the Wnt/β-catenin and the Hh pathways by genetically depleting head or GZ by RNAi treatments. This approach opens up the possibility of identifying target genes in specific regions of embryos that are too small for manual dissection. However, it should be noted that our experiment cannot distinguish between direct or indirect targets. Unexpectedly, we found few targets in the head for either pathway, although these domains correspond to the vertebrate midbrain-hindbrain boundary and the expression of several highly conserved *Tribolium* head patterning genes is initiated there (Posnien et al., 2011). Hence, synergism does not appear to play a dominant role. An alternative reason could be that head patterning lags behind GZ patterning as indicated by the later expression of *Tc-wg* compared with the GZ (Fig. 7; supplementary material Fig. S1). Repeating the same experiment at a later developmental stage might then reveal the targets more faithfully. It remains unclear why the Hh pathway activated so few target genes despite its clear upstream role in the head.

### Wnt integrates pattern formation, cell division and metabolism in the GZ

Although the involvement of the Wnt/β-catenin pathway in posterior development in arthropods is well established, which aspects of regulation are conserved or diverged remains an open question (Martin and Kimelman, 2009). Our dataset offers the first comprehensive view of the target gene sets of both pathways, providing a framework for comparisons with similar target gene sets that await identification in other arthropods. A similar number of genes was regulated by Hh and Wnt/β-catenin signaling in the GZ and the gene sets were almost non-overlapping. The finding of several epidermal patterning genes in the Wnt set of the GZ was not unexpected given the conserved role of Wnt/β-catenin signaling in bilaterian posterior patterning and the known role in activating the pair-rule genes *Tc-eve* and *Tc-runt* in *Tribolium* (Bolognesi et al., 2009; Martin and Kimelman, 2009). We identified several additional pair-rule genes and *Tc-caudal.* Together, this clearly places Wnt/β-catenin signaling at the top of the GZ gene hierarchy.

Surprisingly, we find mesoderm to be under the control of Wnt/β-catenin signaling. This is in contrast to *Drosophila*, in which *twist* is activated by *dorsal* and where the *dorsal*/*snail*/*twist* network activates *wntD* expression (Ganguly et al., 2005).

Wnt/β-catenin (but not Hh) signaling activated a large number of ribosomal genes and genes involved in cell division, indicating that Wnt/β-catenin signaling integrates pattern formation and posterior elongation with metabolism. Recently, it was shown that PYGO, a transcriptional co-activator of *armadillo*/*β-catenin* (Kramps et al., 2002), is involved in ribosome biogenesis in human cancer cell lines, indicating that this might be a common theme of Wnt/β-catenin signaling (Andrews et al., 2013).

With respect to posterior elongation, it remains unknown what the relative contributions of convergent extension and cell division might be. A posterior zone of localized cell proliferation does not appear to exist and, in line with this, Sarrazin et al. (2012) have shown that convergent extension takes place in the GZ. Further, they show that there is a peak of cell division in the GZ in mid-elongating embryos. Our EdU results confirm a significant degree of cell division during mid-elongation.

An intriguing feature of the Hh dataset is the large number of genes predicted to be involved in a proteolytic cascade, including a member of the spätzle family (*Tc-spz1*), which might act as a Toll ligand. Based on these two findings, we hypothesize that the GZ signaling center may act in ongoing dorsoventral patterning, which is required for continuous mesoderm and neuroectoderm formation during elongation (Lynch and Roth, 2011).

### Have most posterior patterning genes been identified?

So far, candidate genes have been studied in terms of their function in GZ patterning in *Tribolium*. Our data contained several crucial segmentation genes (*Tc-caudal*, *Tc-eve*, *Tc-odd*), which suggests that the approach had the power to identify novel patterning genes independently of previous knowledge. Regarding the rather specific posterior expression of the investigated target genes, it was surprising that we did not detect segmentation phenotypes. Posterior patterning genes could still be included in the set of empty egg phenotypes but, given the strong phenotype, it is more likely that these genes perform rather basic cellular functions such as cell division or metabolism. The latter possibility is supported by the GO term analysis of the Wnt pathway, which indicates enrichment in protein biosynthesis and cell division. Taken together, our data suggest that the candidate gene approach of the past decades has already revealed most, if not all, Wnt and Hh target genes relevant to posterior patterning.

### *Tc-sens* acts early in hindgut formation

We found several hindgut genes to be controlled by Wnt signaling in the GZ (Lengyel and Iwaki, 2002) and identify *Tc-sens* as novel hindgut gene. This function is very different from the *Drosophila* ortholog *senseless*, which is not expressed in the hindgut but is essential for sensory organ development (Nolo et al., 2000). Interestingly, its paralog *senseless-2* is expressed in the anterior midgut in *Drosophila* but a phenotype has not described so far. It is intriguing that *Tc-sens* is required in the GZ of the germ rudiment despite the fact that the hindgut differentiates only many hours later. Apparently, there is molecular specification of the hindgut anlagen in the GZ at early stages of abdominal segmentation.

## MATERIALS AND METHODS

### Strains and sequences

*San Bernardino* wild-type strain of *Tribolium castaneum* was used for all experiments and kept at 32°C on white flour supplemented with 5% dry yeast. *Black* males were used for mating females injected with dsRNA. A complete list of primers and sequences is provided in supplementary material Table S1.

### RNAi

dsRNA was synthesized as described previously (Posnien et al., 2009). Lithium precipitation was used for templates longer then 400 bp and phenol/chloroform extraction followed by isopropanol precipitation for shorter templates. dsRNA was injected into female pupae (RNA-seq) or adults (downstream candidates) at 32°C using the following concentrations: *arrow*, 2.1 µg/µl; *frizzled1/2*, 2.3 µg/µl; *hh*, 2.0 µg/µl; *orthodenticle*, 2.4 µg/µl; *torso*, 2.3 µg/µl; *wntless*, 2.1 µg/µl and >2 µg/µl for downstream candidates. All genes knocked down in the RNAi–RNA-seq procedure were published previously with no off-target effects reported (Beermann et al., 2011; Bolognesi et al., 2008; Bolognesi et al., 2009; Farzana and Brown, 2008; Kotkamp et al., 2010; Schoppmeier and Schröder, 2005). The phenotype of *Tc-sens* was confirmed with non-overlapping fragments (see supplementary material Table S1 for sequences).

### RNA isolation and sequencing

RNA of 10- to 11-h-old RNAi and wild-type embryos was extracted using Trizol (Ambion) according to the manufacturer's protocol, followed by digestion with Turbo DNase (Ambion) and phenol/chloroform extraction (Ambion, pH 6.9). Three biological replicates were sequenced (see supplementary material Table S1 for statistics), with each biological replicate consisting of ∼100 pooled embryos (∼15 µl). The RNAi knockdowns resulted in a transcript reduction of RNA-seq reads of the targeted genes by 80-90%, except for *Tc-frizzled2* with 40% reduction. Cuticle analysis of sibling animals confirmed the high penetrance of the RNAi treatments (see Fig. 3 for quality controls). A gene was considered downregulated if the transcript number was reduced by half at a false discovery rate below 0.1 (see MA plots in supplementary material Fig. S3A; datasets in supplementary material Table S2). Principal component analysis (PCA) revealed that the respective treatments cluster (Fig. 3C).

### RNA-seq analysis

The FASTQ formatted Illumina reads were mapped to the *Tribolium* au2 gene set (http://bioinf.uni-greifswald.de/tcas/genes/au2/) using bowtie2 (Langmead and Salzberg, 2012) with the ‘very sensitive’ presetting. Reads were counted with SAMtools (Li et al., 2009) and combined in a counts table. Statistical analysis of the data and differentially expressed gene calling was performed in R (http://www.r-project.org/) using the DESeq package (Anders and Huber, 2010) from Bioconductor (Gentleman et al., 2004). Genes were considered to be differentially expressed if log2 fold change was ≥1 given an adjusted *P*-value of <0.1. For exploratory clustering analysis the variance stabilizing normalization was used and PCA was performed using the function plotPCA. Before PCA, all genes with counts of less than 500 in two or more experiments were filtered out and only the top 500 remaining genes, which display the highest overall variances, were used. Intersects and Venn diagrams were built with the Overlapper.R function (http://manuals.bioinformatics.ucr.edu/home/R_BioCondManual#TOC-Venn-Diagrams). Heatmaps were plotted with gplots (http://cran.r-project.org/web/packages/gplots/index.html) and RColorBrewer (http://cran.r-project.org/web/packages/RColorBrewer/index.html), bar charts with ggplot2 (Wickham, 2009). MA plots and sample clustering heat maps were made with the DESeq package.

### Annotation of RNA-seq results

The au2 gene set was BLASTed against the gene set from FlyBase (Gelbart et al., 1997) using BLAST (Altschul et al., 1997) implemented in BioPerl (Stajich et al., 2002) and a custom perl script (http://bioinf.uni-greifswald.de/bioinf/bioinfprakt11/ex3/orthoparahomlist.pl). Reciprocal best BLAST scores were reported as orthologs. Hits with an E-value <10^−5^ were considered homologs and included in case no ortholog was found. The resulting list of au2 gene IDs with their corresponding Fbpp ID was merged to the *Drosophila* annotations from FlyBase in R. GO term enrichment was performed with AmiGO using the orthologs/homologs from the above list and FlyBase as database filter (Carbon et al., 2009).

### *In situ* hybridization

*In situ* hybridization was performed as described previously (Schinko et al., 2009) with minor changes: Roche Blocking Reagent was added to the HybeA buffer and used as the standard blocking buffer in all following steps. PBT was substituted with maleic acid buffer [100 mM maleic acid (pH 7.5), 150 mM NaCl, 0.1% Tween20] as the standard washing buffer. Fluorescence *in situ* hybridization was performed by adapting the zebrafish protocol (Lauter et al., 2011) to *Tribolium*.

### EdU cell proliferation assay

The EdU cell proliferation assay was performed with the Click-iT EdU Alexa Fluor 488 Imaging Kit according to the manufacturer's protocol (Life Technologies). Embryos were 10-14 h AEL (32°C) during EdU injections and incubated for a further 3 h (32°C) prior to fixation and EdU staining. The embryos were counterstained with Hoechst 33342.

### Microscopy and imaging

Cuticles were imaged on a Zeiss LSM780 with a 25× objective using a 550 nm laser. The resulting stacks were processed in Amira 5.32 (FEI). Four steps of blind deconvolution and Gaussian smoothing were applied. Intensity levels were set to range from 5-200. *In situ* stainings of germ bands were imaged on a Zeiss Axioplan2, 10× objective, using ImagePro 6.2 software (Media Cybernetics). Blastoderm *in situ* stainings were recorded as 8-bit mono for enhanced signal to overcome quenching and epifluorescence of the yolk. EdU-stained embryos were imaged on a Zeiss LSM780 with a 10× objective using 405 and 480 nm lasers. The resulting stacks were loaded in Amira 5.32 and 3D rendered with the voltex module. All images were assembled in Photoshop CS2 (Adobe). All figures were imported into Inkscape (http://www.inkscape.org/) for labeling and formatting.

### Data access

The complete dataset, including all relevant parameters, was deposited at GEO under accession number GSE54706.

## Supplementary Material

Supplementary Material
