# Supplementary material for: Wnt/β-catenin signaling integrates patterning and metabolism of the insect growth zone
Source: Development. 2014 Dec 15;141(24):4740–50. doi: 10.1242/dev.112797 (PMC4299277; doi:10.1242/dev.112797)
Supplement: Supplementary Material [file supp_141_24_4740__index.html]

Supplementary Material 

# Wnt/β-catenin signaling integrates patterning and metabolism of the insect growth zone

## DEV112797 Supplementary Material

**Files in this Data Supplement:**

- Supplementary Material
