# Supplementary material for: Wnt/β-catenin signaling integrates patterning and metabolism of the insect growth zone
Source: Development. 2014 Dec 15;141(24):4740–50. doi: 10.1242/dev.112797 (PMC4299277; doi:10.1242/dev.112797)
Supplement: Supplementary Material [file supp_dev.112797_DEV112797supp.pdf]

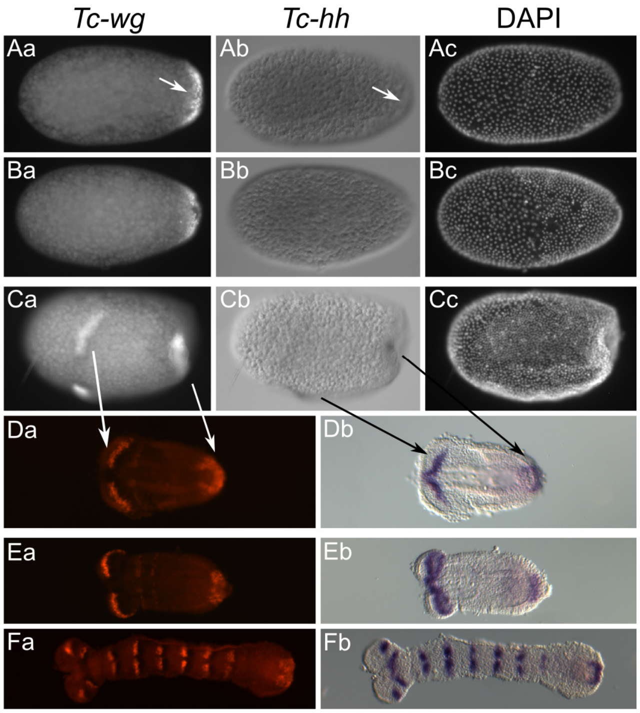


**Fig. S1 – Expression dynamics of *Tc-wg* and *Tc-hh***
Developmental time series of *Tc-wg* (Aa-Fa) and *Tc-hh* (Ab-Fb) double stainings and respective DAPI stainings (Ac-Cc). *Tc-wg* expression was initiated in the late undifferentiated blastoderm at the posterior pole (white arrow in Aa). After the germ rudiment had formed *Tc-wg* was additionally expressed at the ocular parasegment boundary in the head region (Ca-Da). At this stage *Tc-hh* expression was also initiated at the ocular parasegment as well as in the GZ (Cb-Db). The stage shown in rows C and D were used in the RNAseq approach and corresponded to 10-11h old embryos (at 32°C).

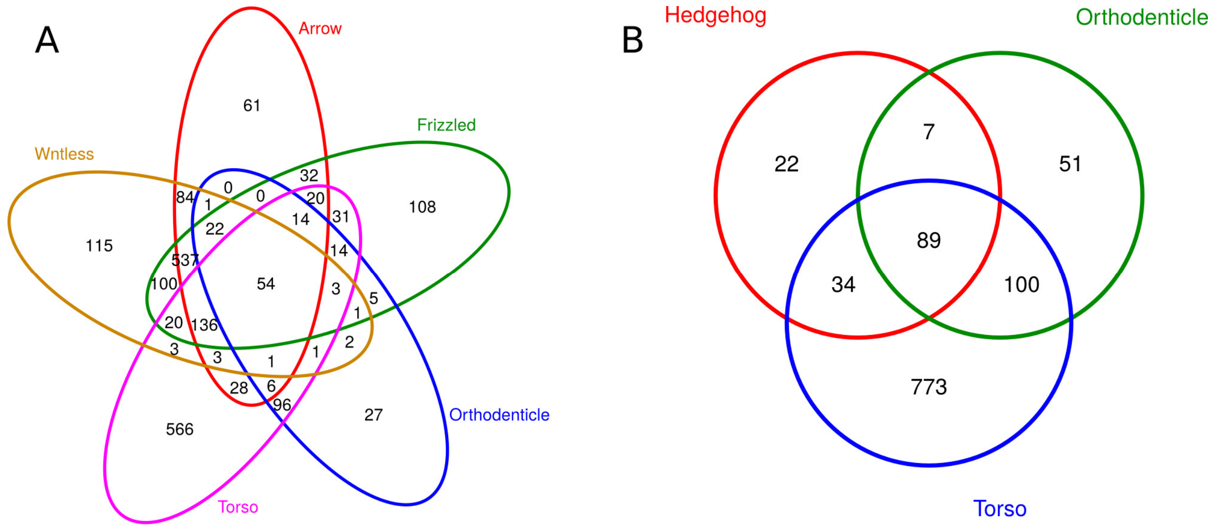


C

| Venn Intersect | # candidates | # with low Counts (<200) | % with low Counts (<200) |
|---|---|---|---|
| Anterior Wnt UP | 22 | 20 | 90.9 |
| Anterior Wnt DOWN | 8 | 2 | 25.0 |
| Anterior HH UP | 7 | 3 | 42.9 |
| Anterior HH DOWN | 8 | 1 | 12.5 |
| Posterior Wnt UP | 136 | 95 | 69.9 |
| Posterior Wnt DOWN | 72 | 6 | 8.3 |
| Posterior HH UP | 34 | 27 | 79.4 |
| Posterior HH DOWN | 87 | 14 | 16.1 |

**Fig. S2 – Venn upregulated genes**
(A) Venn diagram of upregulated Wnt treatment genes. 136 genes in the posterior Venn intersect and 22 genes in the anterior Venn intersect were found. (B) Venn diagram of upregulated Hh treatment genes. 34 genes in the posterior Venn intersect and 7 genes in the anterior Venn intersect were found. (C) Table comparing the number of up- and downregulated in the different Venn intersects. Among the upregulated genes a much larger precentage had very low count numbers when compared to the downregulated genes.

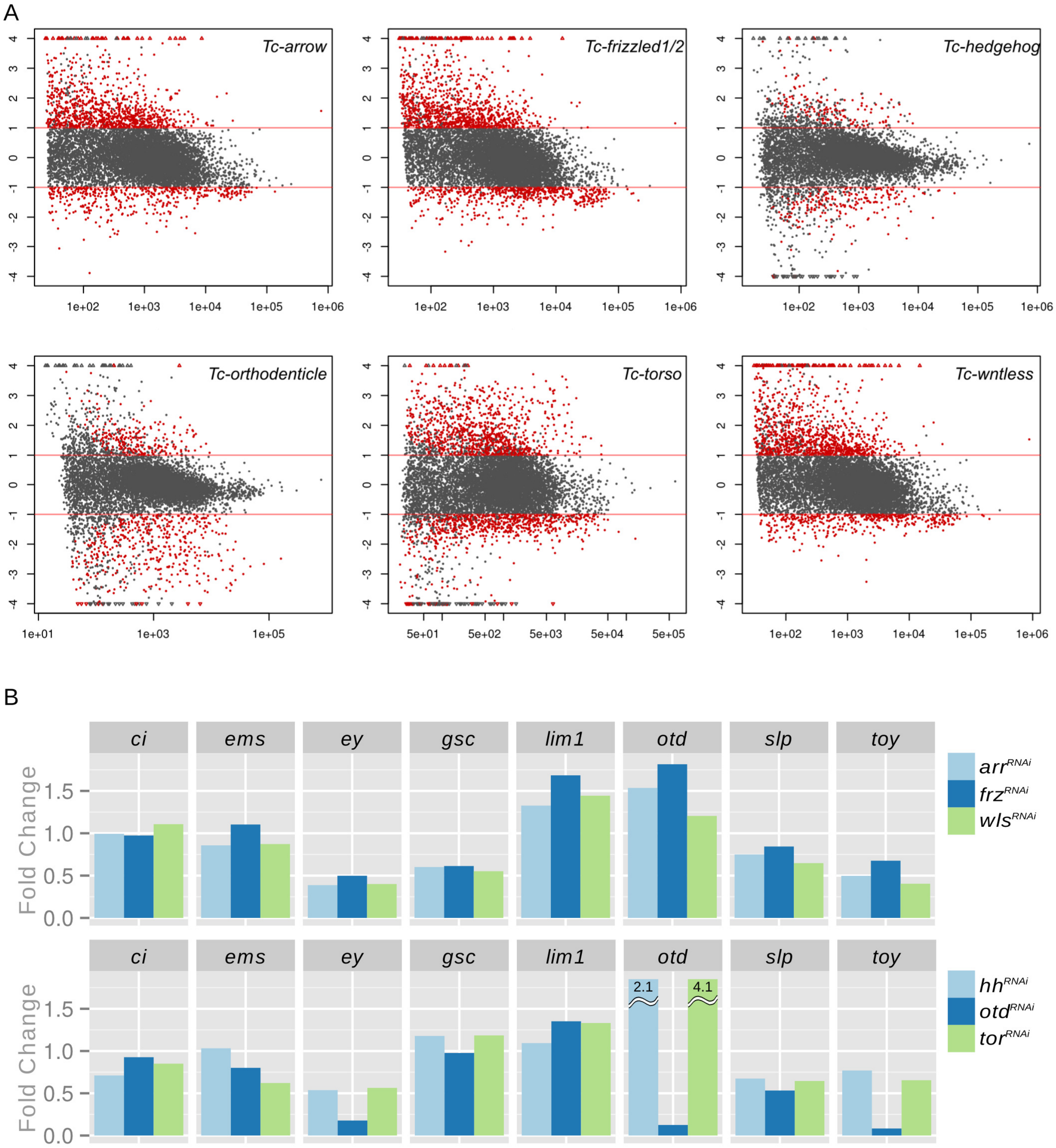


**Fig. S3 - MA plots of RNA-seq treatments and barplots of head candidate genes**
(A) MA plots of the expression differences between RNAi treatments and wildtype. Genes with significantly altered expression are shown in red (false discovery rate < 0.1). Y-axis: log2 fold

change values. X-axis: Mean normalized counts of treatment and wild type samples. (B) Read counts of genes that were expected to be regulated in the head. See discussion for details.

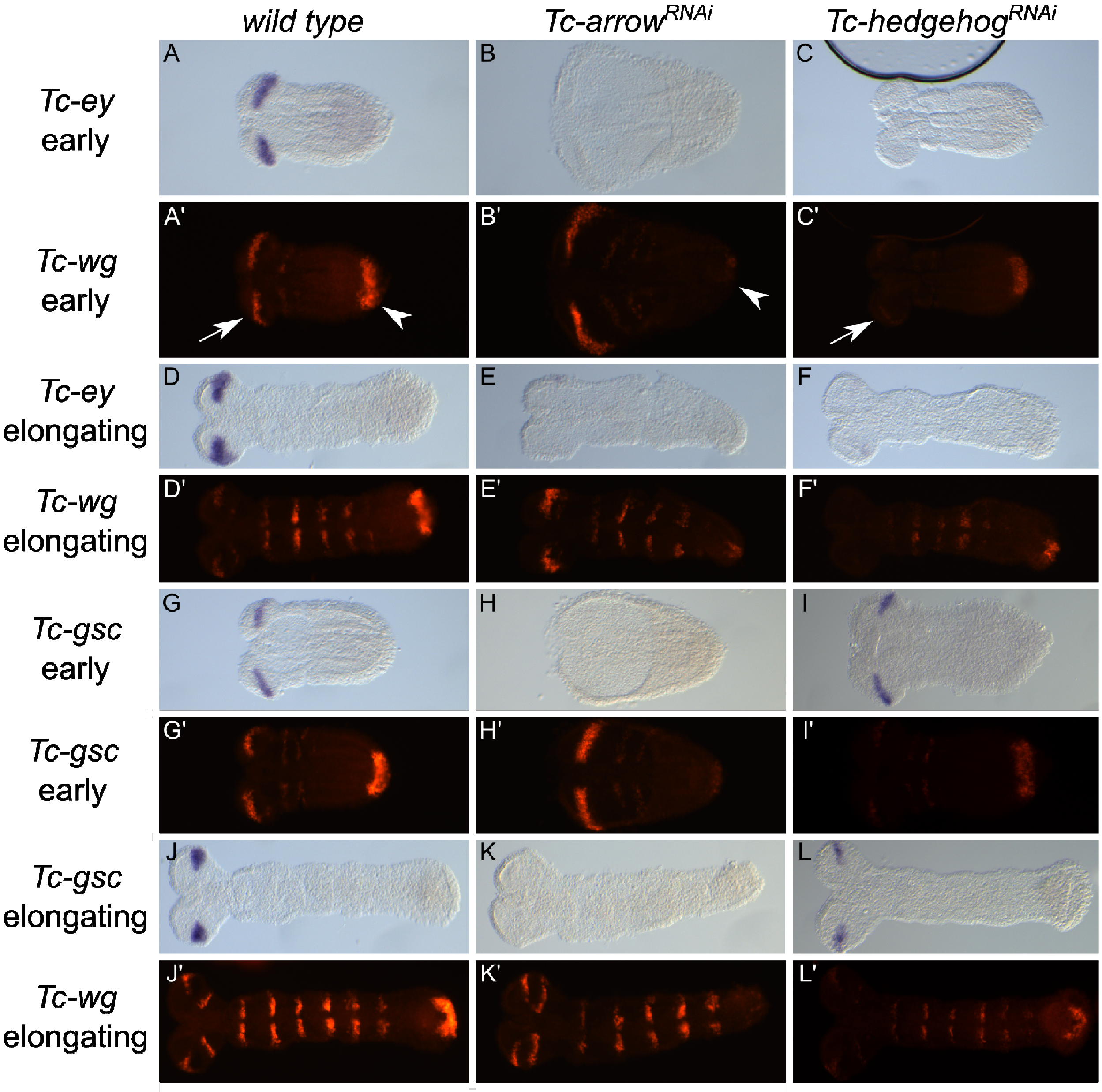


**Fig. S4 – Staining of anterior target genes in *Tc-hh*$^{RNAi}$ and *Tc-arr*$^{RNAi}$**
(A-F) Expression of *Tc-ey* in wild type (A, D), *Tc-arr*$^{RNAi}$ (B, E) and *Tc-hh*$^{RNAi}$ (C, F). *Tc-ey* expression is lost in both RNAi treatments. Counter staining for *Tc-wg* confirmed successful knockdown (A'-F'): In wild type embryos, ocular *Tc-wg* expression (white arrow in A') and GZ *Tc-wg* expression (white arrow head in A') show comparable signal intensity. In *Tc-arr*$^{RNAi}$ the GZ *Tc-wg* domain is lost or strongly reduced (white arrow head in B', E') with the ocular *Tc-wg* domain unaffected. In *Tc-hh*$^{RNAi}$ the ocular *Tc-wg* domain is lost or strongly reduced (white arrow in C', F') with GZ *Tc-wg* unaffected. See also Fig.1 for *Tc-wg* expression in *Tc-arr*$^{RNAi}$ and *Tc-hh*$^{RNAi}$. (G-L) Expression of *Tc-gsc* in wild type (G, J), *Tc-arr*$^{RNAi}$ (H, K) and *Tc-hh*$^{RNAi}$ (I, L). *Tc-gsc* expression is lost in *Tc-arr*$^{RNAi}$ but not in *Tc-hh*$^{RNAi}$. (G'-L') Internal *Tc-wg* control stainings show the strength of the RNAi phenotypes.

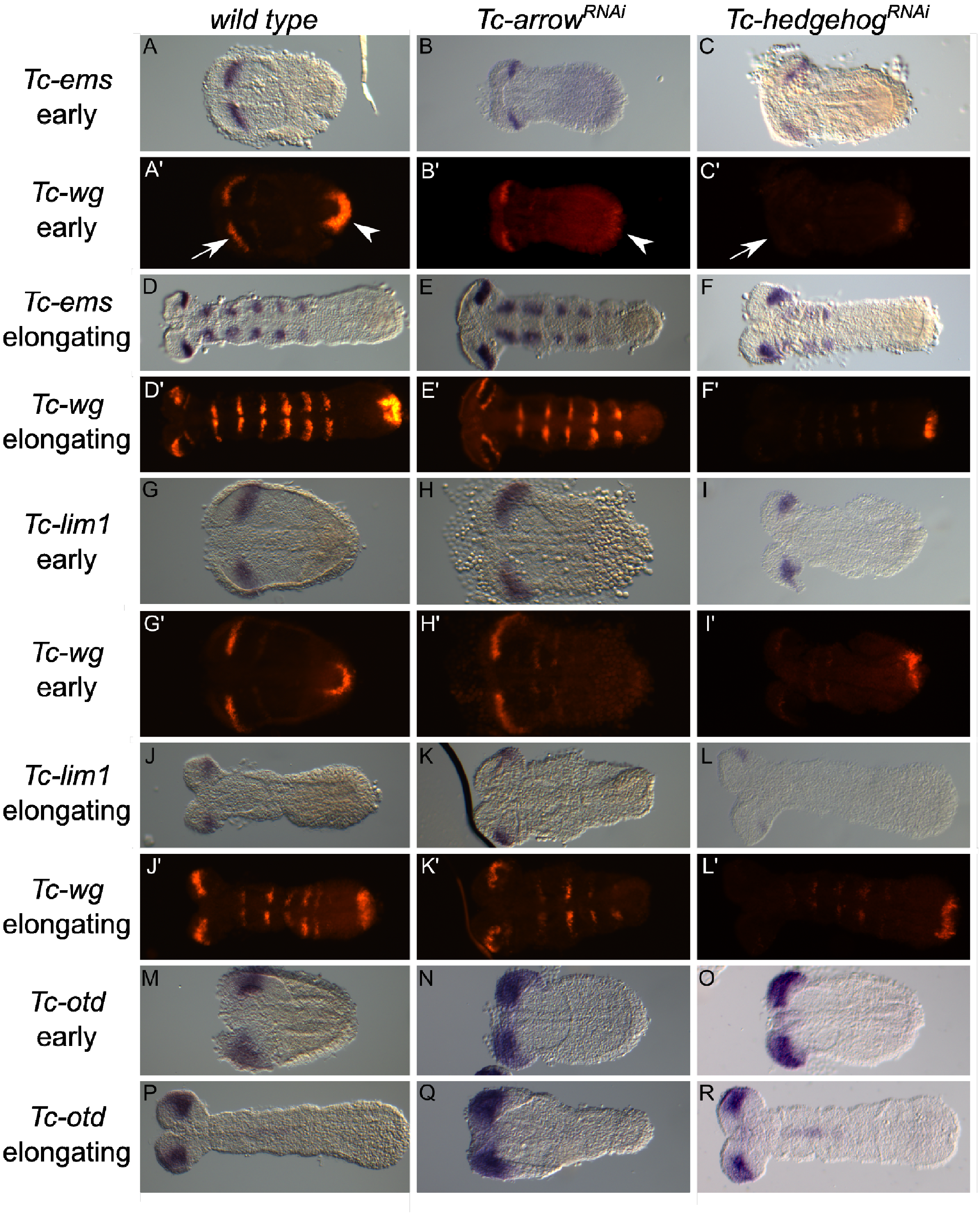


**Fig. S5 – Staining of non-target genes in *Tc-hh*$^{RNAi}$ and *Tc-arr*$^{RNAi}$**
(A-F) Expression of *Tc-ems* in wild type (A, D), *Tc-arr*$^{RNAi}$ (B, E) and *Tc-hh*$^{RNAi}$ (C, F). *Tc-ems* expression is unaffected in *Tc-arr*$^{RNAi}$ and *Tc-hh*$^{RNAi}$. (G-L) Expression of *Tc-lim1* in wild type (G, J), *Tc-arr*$^{RNAi}$ (H, K) and *Tc-hh*$^{RNAi}$ (I, L). *Tc-lim1* expression is unaffected in *Tc-arr*$^{RNAi}$ and *Tc-*

*hh*$^{RNAi}$. (M-R) Expression of *Tc-otd* in wild type (M, P), *Tc-arr*$^{RNAi}$ (N, Q) and *Tc-hh*$^{RNAi}$ (O, R). *Tc-otd* expression is unaffected in *Tc-arr*$^{RNAi}$ and *Tc-hh*$^{RNAi}$. (A'-L') Counter staining for *Tc-wg* confirmed successful knockdown. In wild type embryos, ocular *Tc-wg* expression (white arrow in A') and GZ *Tc-wg* expression (white arrowhead in A') show comparable signal intensity. In *Tc-arr*$^{RNAi}$ the GZ *Tc-wg* domain is lost or strongly reduced (white arrow head in B') with the ocular *Tc-wg* domain remaining unaffected. In *Tc-hh*$^{RNAi}$ the ocular *Tc-wg* domain is lost or strongly reduced (white arrow in C') with GZ *Tc-wg* unaffected. This corresponds to the results shown in Fig.1 and, hence, confirm successful knockdown in the stained embryos. *Tc-wg* control staining was not performed in *Tc-otd* in situs. Cuticle analysis of sibling animals and the altered embryo morphology (e.g. in Q) indicate that the shown embryos are affected by the RNAi treatment.

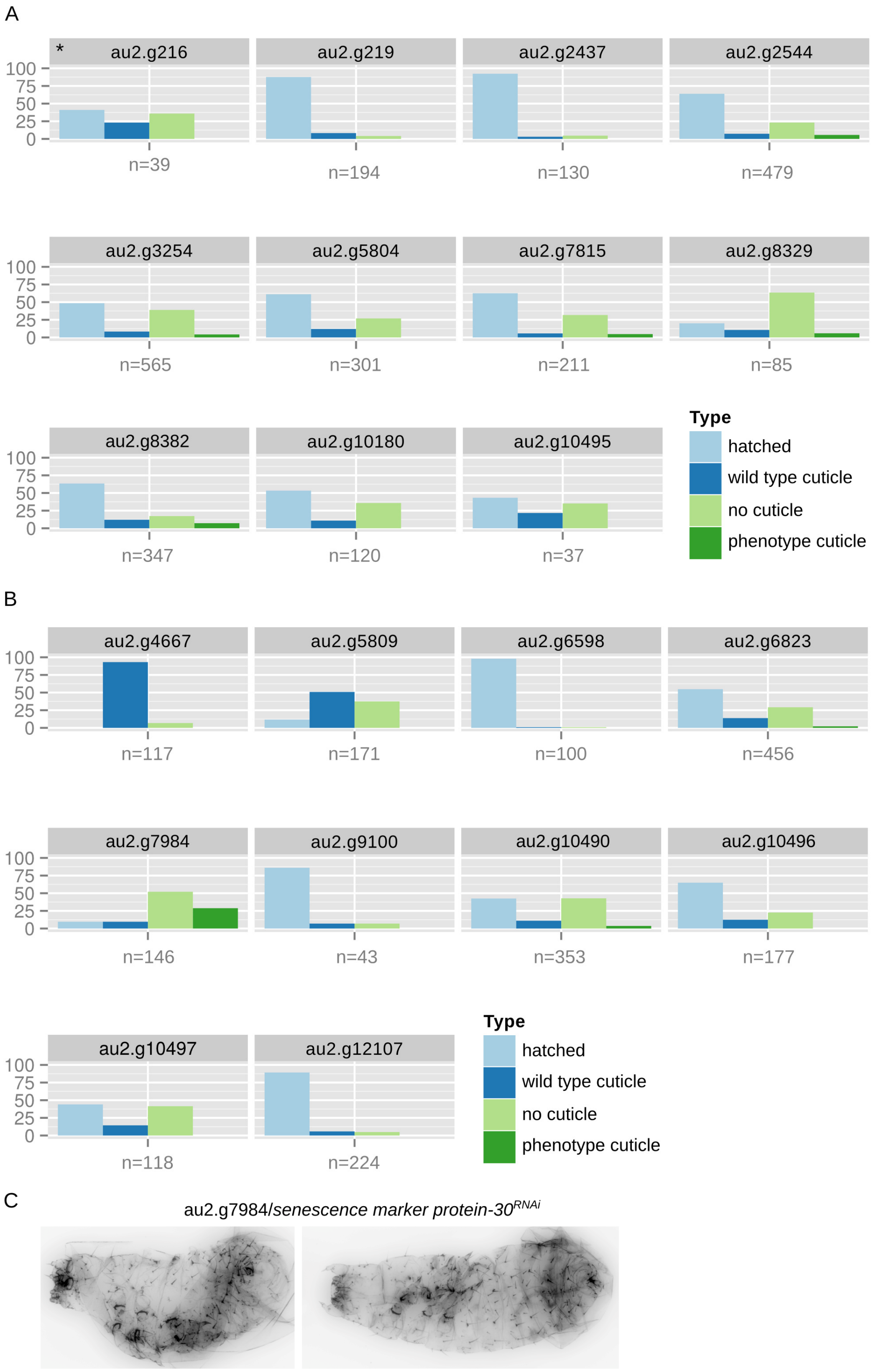


**Fig. S6 - Quantification of RNAi cuticle phenotypes of selected candidate genes**
Barplots showing percentages of phenotype categories, i.e hatched, wild type cuticle (not hatched

but no cuticular defects), no cuticle (empty egg) and cuticles with morphological phenotypes. (A) Posterior Wnt candidates: 14 dsRNA injections were performed. One injections killed all animals (au2.g11365/*Rpb12*) and one led to sterility (au2.g8732). The phenotype of au2.g7373/*sens* is described in detail in Fig. 6. The remaining eleven genes are shown. Gene au2.g216 (*also candidate in Hh set) resulted in a low egg-lay rate. Genes au2.g3254, au2.g7815, au2.g8329, au2.g10180 and au2.g10495 showed a high percentage (>30%) of empty eggs (no cuticle produced). Only a very small percentage of cuticles showed severe but unspecific defects in various treatments (dark green bars) and are not considered to be significant. (B) Posterior Hh candidates: Ten dsRNA injections were performed. One gene was already included in the Wnt set (au2.g216, shown above). au2.g9100/*pwn* led to a reduced egg-lay rate. The genes au2.g6823, au2.g10490/*IA-2* and au2.g10497 resulted in increased empty eggs. The two candidates au2.g4667/*LanB2* and au2.g5809/*ndg* resulted in very low hatch rates withoug cuticular defects. au2.g7984/*smp-30* resulted in 50% empty eggs and 29% showing the phenotype described below. (C) au2.g7984 dsRNA cuticle phenotype. Lateral and ventral views of RNAi cuticles showing disturbed patterning of all segments and appendages. The posterior segments do not appear to be more affected than the anterior ones. Many cuticles were dorsally not closed and/or bent dorsally indicating problems with dorsal closure.

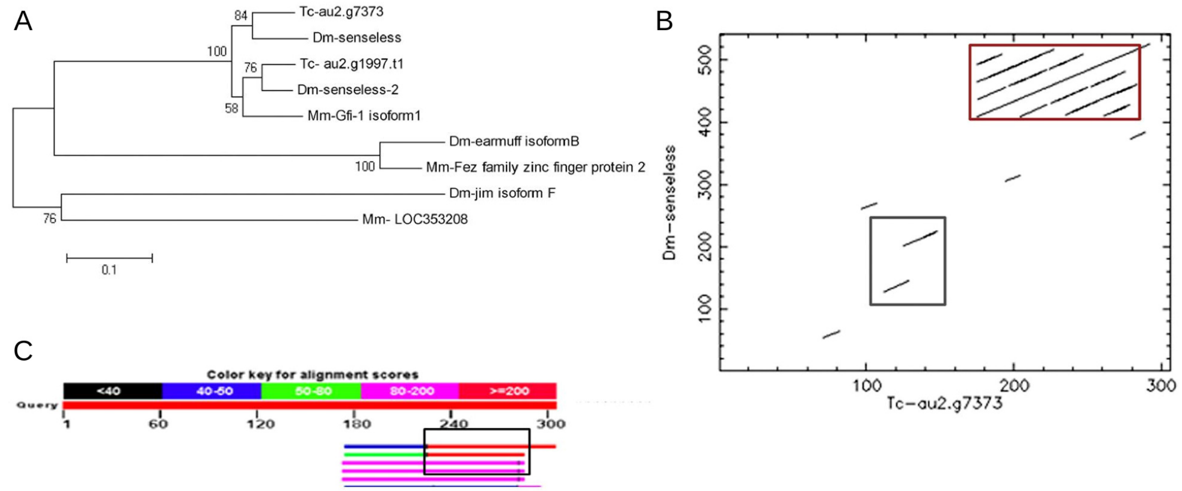


**Fig. S7 - Neighbour joining tree and DotmatcherP plot reveals orthology of Tc013474 with *Tc-Senseless***

BlastP with Tc013474 (annotation version au2.g7373) was performed in *Drosophila* and Mouse RefSeq protein collections. The best hits were aligned with ClustalW as implemented in Mega5 with standard settings. Only residues present in all proteins were used for phylogenetic analysis. Maximum Parsimony, Neighbour Joining and Maximum Likelihood gave comparable relationships where Tc013474 clustered with *Drosophila* Senseless and the new annotation au2.g1997 consisting of the fused annotation of the OGS 3 genes Tc012906/Tc012905 clustered with Senseless-2 (see neighbor joining tree in panel A). This orthology was further confirmed by three additional regions of similarity N-terminal of the zinc fingers and the number of zinc finger domains (4 for Senseless and 6 for Senseless-2) revealed by Prosite domain scanning (Expasy). The region with the zinc finger domains is marked with a red box in the DotmatcherP plot (EBI) in panel B. Gfi-1 was the only mouse protein on this branch of the tree and did not clearly cluster with either the insect Senseless or Senseless-2 orthologs. One additional conserved domain was found in Mouse Gfi-1 and the insect Senseless orthologs (one domain in *Tribolium*, split domain in *Drosophila*; see grey box in panel B). It should be noted that the two N-terminal zinc finger motifs were strongly diverged in both *Drosophila* Senseless and Senseless-2 but rather conserved between *Tribolium* and Mouse (see panel C).

**Table S1. Primer sequences, GO enrichment analysis and mapping statistics**

Click here to Download Table S1

**Table S2. Downregulated genes and Venn intersects**

Click here to Download Table S2

**Table S3. Upregulated genes and Venn intersects**

Click here to Download Table S3
